# Effectiveness and Key Success Factors for Implementation of Problem-Based Learning in Debre Tabor University: A Mixed Methods Study

**DOI:** 10.4314/ejhs.v30i5.21

**Published:** 2020-09

**Authors:** Awoke Wondie, Tegbar Yigzaw, Solomon Worku

**Affiliations:** 1 Reproductiive Health, Debretabor University, Amhara Regional State, Ethiopia; 2 JHPIEGO, Resources for Health, Addis Ababa, Ethiopia; 3 Human Resources Directorate, Federal Ministry of Health, Addis Ababa, Ethiopia

**Keywords:** Problem-based learning, Implementation, Process, Perception, Ethiopia

## Abstract

**Background:**

Problem-based learning has been adopted as a core educational strategy for education of health professionals in more than a dozen of higher education institutions in Ethiopia. Debre Tabor University College of Health Sciences (DTUCHS) is one of the adopters. However, its effectiveness has not been researched yet. Thus, the objective of this study is to assess the quality of PBL implementation, its effectiveness in developing desired student learning outcomes and factors that facilitate or impede PBL implementation.

**Methods:**

A cross-sectional study was conducted in DTUCHS from May to June 2018. We collected quantitative data from students and tutors using self-administered questionnaire. We complemented this with key informant interviews with academic leaders. We computed descriptive statistics from quantitative data while qualitative data were subjected to thematic analysis.

**Results:**

A total of 308 students, 42 tutors and 8 academic leaders were included in the study. Students, tutors and academic leaders perceived that PBL was effective in developing knowledge, problem-solving skills, self-directed learning skills and collaboration competencies. The implementation process showed the existence of clear objectives, appropriate cases, and reasonable workload. Students rated tutors' performance positively, and tutors also rated student learning affirmatively. However, unlike tutors, students thought that the assessment of student performance in PBL was not appropriate. The factors that facilitated PBL implementation were students' and tutors' buy-in, clear curriculum design, adequate infrastructure, commitment to hire more faculty and develop their teaching skills continuously and strong coordination and monitoring.

**Conclusion:**

The findings of our study support the introduction of PBL in a resource-constrained setting. Students, tutors and academic leaders perceived PBL to be effective in achieving desired student learning outcomes. Its implementation was considered consistent with the principles of PBL. Respondents identified the presence of enabling factors to implement PBL in Debre Tabor University (DTU).

## Introduction

Problem-based learning (PBL) is an educational strategy in which “problems are used as a trigger for learning; students collaborate in small groups; learning takes place under the guidance of a tutor; learning is student-initiated; and the curriculum includes ample time for self-study” (1). PBL curriculum was pioneered by the Faculty of Medicine at McMaster University in 1968 due to dissatisfaction with the observation that medical students did not retain and apply the basic science knowledge by the time they enter clerkship. PBL has since spread throughout the world transcending disciplines and the primary-secondary-tertiary education divide (2).

Proponents of PBL argue that it facilitates comprehension and long-term retention of knowledge. Problems and small group discussions in PBL encourage activation and elaboration of prior knowledge which increases motivation to learn because problems arouse situational interest (1). However, the effectiveness of PBL compared to lecture-based curriculum in improving learning outcomes has been a subject of several studies and debates (3). A recent systematic review of the effects of PBL on physician competence after graduation found that physicians trained through PBL were better at coping with uncertainty, appreciating legal and ethical aspects of healthcare, communication skills and continuing self-directed learning than those trained with traditional education strategy. However, the results were inconclusive when it came to knowledge outcomes, indicating that PBL is not a panacea (4). Some argue that the lack of efficacy of PBL is due to the varied understanding and practice of the method in different contexts (2). Importantly though, there is a growing realization that combining PBL with other teaching/learning methods may yield better results (3).

Despite the rapid acceptance, many attempts to use PBL have failed to reach the results expected. Apart from philosophical objections as an educational method and resistance from faculty, PBL implementation could be affected by many factors that could emanate from the curriculum, institution and students. Ignoring these factors was the major reason for failed PBL implementation in many institutions (5, 6).

PBL as a curriculum concept has a relatively short history in Ethiopia but is becoming increasingly important. PBL was adopted as an educational strategy in the 2011 innovative medical curriculum, which is being implemented in 13 medical schools around the country (7). The College of Health Sciences at Debre Tabor University (DTUCHS) also adopted PBL as one of the key educational strategies when it designed innovative curricula for medicine and midwifery in 2013 as well as other disciplines in subsequent years (8). PBL is also integrated into recently developed national curricula for different health disciplines, indicating a trend towards more widespread adoption in Ethiopia. Given its growing importance in health science curricula, it is high time that a study is done to evaluate whether PBL results in better learning outcomes. Moreover, although context has an important impact on the success or failure of PBL (2), we have not come across a local study on PBL implementation. Therefore, this study aimed to assess the quality of PBL implementation, its effectiveness in developing desired student learning outcomes and the factors that facilitate or impede PBL implementation.

## Methods

**Study design and period**: A cross-sectional study that combines a self-administered questionnaire and qualitative interviews was conducted between May and June 2018.

**Study setting**: The study was done in Debre Tabor University College of Health Sciences (DTUCHS). DTUCHS designed and implemented innovative curricula that combines PBL along with other contemporary teaching/learning methods. The curricula suggest that a group of 5 to 8 students meet twice per week to complete one PBL case in a week. In the first meeting, learners identify problems, generate hypotheses and explain mechanisms with gradual disclosure of information. Days between the two sessions are dedicated to self-study on learning issues identified. During the second meeting, students discuss the learning issues to apply what they have learned to the problem and synthesize the learning agendas addressed. Faculty from clinical, biomedical and public health disciplines develop and/or review PBL cases before the next academic year begins. This study focused on departments of medicine and midwifery that have a longer history of PBL implementation than the others. Both medical and midwifery students have PBL sessions every week starting from the second semester of the first year through the final year.

**Population and sampling**: The study populations for the self-administered questionnaire were students and tutors while we targeted academic leaders for the key informant interviews. At the time of the study, there were 220 medical students from year one through year five and 88 midwifery students in second and third years. However, first year midwifery students had not started implementing PBL during data collection and were thus excluded from this study.

At the time of data collection, there were 30 academic staff in the Department of Medicine and 26 in the Department of Midwifery, and we sought to cover all those who met the inclusion criteria. The inclusion criteria for academic staff were participating in PBL case development and/or tutoring for at least one academic year. The college had 13 academic leadership positions at the time of the study. Academic leaders (deans, department heads and coordinators) with experience of 12 months or more were purposively selected for the qualitative interviews. Qualitative interviews continued until a point of saturation was reached.

### Data Collection

**Quantitative data**: Self-administered questionnaire was used to obtain students' and tutors' perspectives on effectiveness of PBL (17 items arranged in 6 main themes), process of PBL implementation (14 items for students and 13 items for tutors, both arranged in 5 main themes), student performance (17 items arranged in 6 main themes), tutor performance (13 items arranged in 5 main themes) and barriers for PBL implementation (9 items for students and 8 items for tutors). The response options for the first four variables were a 5-point Likert scale: 5 = strongly agree, 4 = agree, 3 = neutral, 2 = disagree, and 1= strongly disagree. The response options for the barriers of implementation were a four-point Likert scale: 1 = not a barrier, 2 = somewhat a barrier, 3 = moderate barrier and 4 = extreme barrier. The items in the questionnaire were adapted from previous studies (9–14).

**Qualitative data**: Semi-structured interviews were conducted to capture the views of academic leaders regarding the effectiveness of PBL, its implementation and the factors that facilitate or impede PBL implementation. The interview was tape-recorded and completed within an average of 25 minutes. The lead author conducted all the interviews.

**Validity and reliability**: The data collection tools for tutors, students and academic leaders were pilot-tested with 9 tutors, 11 students and 3 academic leaders, respectively. Participants for the pilot-test were not from the departments of medicine and midwifery. Appropriate modifications were made to the tools following the pilot, which included writing more clear instructions, editing some items and removing items that were not aligned with the objectives of the study. The reliability of quantitative tools was estimated using the Cronbach's coefficient alpha test, which yielded 96% and 91% for tutors and students, respectively, showing a very high level of internal consistency.

**Data analysis**: The quantitative data were entered and processed using Statistical Package for Social Sciences (SPSS), version 20, to calculate percentage, median and inter-quartile range for each item and the main factors. We merged “Strongly agree” and “Agree” in the narrative for ease of readability. Perceived barriers of implementation were dichotomized and presented as “Barrier” (by merging 1= Not a barrier and 2 = Somewhat a barrier), and “Major barrier” (by merging 3 = Moderate barrier, and 4 = Extreme barrier).

The qualitative interviews were transcribed verbatim by the lead author. Braun and Clarke's six-phase framework for doing a thematic analysis was applied, which includes familiarizing with data, generating initial codes, searching for themes, reviewing themes and defining themes (15). The evaluator coded and categorized the responses, and several themes emerged from them. The evaluator reviewed the interviews again and a point of saturation was ensured in the 8^th^ interview coding when the same themes and sub-themes were identified. The final result was presented by triangulating the quantitative data in a narrative way supported by selected direct quotes.

**Ethics**: This study was approved by Debre Tabor University Research Ethics Committee. All participants were informed of the objective of the study and gave their written consent to participate.

## Results

**Characteristics of participants**: A total of 308 students (220 Medicine and 88 Midwifery) and 42 tutors (18 Medicine and 24 Midwifery) returned a completed questionnaire, which yielded a response rate of 100%. The mean age of students was 20.97±2.07 years while that of tutors was 27.02 +3.17 years (Table 1).

**Table 1:** Characteristics of quantitative study participants among the departments of medicine and midwifery, DTU, 2018

Variable	Faculty		Student				
	
	Range	Mean ± SD	Range		Mean age		
Age	25–40	27.02±3.17	20–26		21.03		
Sex	Male	Female	Male		Female		
	33	9	173		135		
School	Midwifery	Medicine	Midwifery		Medicine		
	24	18	88		220		
Education	Educational Status		Academic year				
	1^st^ Degree	2^nd^ Degree	1^st^ yr	2^nd^ yr	3^rd^ yr	4^th^ yr	5^th^ yr
	32	10	58	90	86	44	30

**Perceived effectiveness of PB**: Students and tutors agreed that PBL facilitated attainment of desired learning outcomes: developing knowledge, problem-solving skills, self-directed learning skills, motivation and group collaboration skills. Promoting group collaboration, improving motivation and constructing professional knowledge were the top three benefits, drawing agreement from 79.3%, 76.9%, and 75.5% of students, respectively. Over half of students (57.3%) also said they preferred PBL over lecture(Table 2). Tutors' perceptions of PBL's educational benefits were even more favourable, with the top three ratings going to promoting group collaboration (95.1%), improving motivation (92.8%) and developing self-directed learning skills (86.3%). Most tutors (73.8%) also said they preferred PBL over lecture (Table 3). The perceived effectiveness of PBL in student learning was also echoed in the qualitative component of the study. Academic leaders reported that realistic scenarios help students to relate concepts to everyday activities, and discussion among them strengthens understanding of subject matter. The following quote from the head of Medicine Department goes:

“PBL enables them to remember what they understand from cases and share what they understand, improving their communication and reasoning skills. So, it was effective in supporting student learning.”

**Table 2:** Students' perception of PBL as a teaching strategy in the departments of medicine and midwifery, DTU, 2018 (N=308)

S.no	Item	SDA n(%)	DA n(%)	N n(%)	A n(%)	SA n(%)	Mdn (IQR)
**Construction of Professional** **knowledge**		**12(3.9)**	**24(7.8)**	**39(12.5)**	**123(39.7)**	**111(35.8)**	**4.00(1.50)**

1	Use and activate my prior knowledge	13(4.2)	21(6.8)	29(9.4)	134(43.4)	111(35.9)	4.00(1)
2	Interpret, analyze, and apply key concepts precisely	9.(2.9)	19(6.1)	37(12)	146(47.2)	97(31.4)	4.00(1)
3	Enhance deep understanding of knowledge	8(2.6)	28(9.1)	38(12.3)	123(39.8)	111(35.9)	4.00(1)
4	Gain and retain knowledge for a long period	19(6.2)	29(9.4)	50(16.2)	87(28.2)	123(40)	4.00(2)
**Development of Problem-solving** **skills**		**15(4.9)**	**30(9.6)**	**53(17.2)**	**141(45.7)**	**69(22.4)**	**4.00 (1)**

5	Increase my ability to solve real-world problems	24 (7.8)	48(15.5)	66(21.4)	137(44.3)	33(10.7)	4.00(1)
6	Encourage me to consider alternatives when solving a problem	10 (3.2)	25(8.1)	49(15.9)	147(47.6)	77(24.9)	4.00(2)
7	Enable me to make reasonable conclusions	12 (3.9)	16(5.2)	44(14.2)	139(45)	97(31.4)	4.00(1)
**Development of Self-directed** **learning**		**19(6.1)**	**32(10)**	**51(16.7)**	**124(40.1)**	**82(26.5)**	**4.00(1.50)**

8	Identify gaps in my knowledge	22(7.1)	25(8.1)	45(14.6)	118(38.2)	98(31.7)	4.00(2)
9	Identify and utilize resources for PBL like a reference, internet	16(5.2)	29(9.4)	42(13.6)	135(43.7)	86(27.8)	4.00(2)
10	Think independently	16(5.2)	28(9.1)	63(20.4)	127(41.1)	74(23.9)	4.00(1)
11	Plan my work	22(7.1)	46(14.9)	56(18.1)	115(37.2)	69(22.3)	4.00(1)
**Improvement of Motivation**		**19(6.1)**	**23(7.6)**	**29(9.4)**	**114(37.0)**	**123(39.9)**	**4.00(1.50)**

12	Learn more	16(5.2)	19(6.1)	24(7.8)	121(39.2)	128(41.4)	4.00(1)
13	Stimulate my interest in learning	21(6.8)	28(9.1)	34(11)	107(34.6)	118(38.2)	4.00(2)
**Promoting group collaboration**		**11(3.4)**	**19(6.1)**	**34(11)**	**135(44)**	**109(35.3)**	**4.00** **(1.00)**

14	Improves my ability to participate in open discussion of differing opinions	9(2.9)	19(6.1)	37(12)	138(44.7)	105(34)	4.00(1)
15	Share what I learned or understand	9(2.9)	12(3.9)	31(10)	135(43.7)	121(39.2)	4.00(1)
16	Respect others opinion	14(4.5)	26(8.4)	34(11)	134(43.4)	100(32.4)	4.00(1)

**I prefer PBL to lecture as an** **effective method of learning**		**40(13)**	**46(15)**	**45(14.6)**	**85(27.5)**	**92(29.8)**	**4.00(3.00)**

SDA= Strongly Disagree, DA= Disagree, N= Neutral, A= Agree, SA= Strongly Agree, Mdn= Median, IQR= Inter- Quartile Range

**Table 3:** Tutors' perception of PBL as a teaching strategy in the departments of medicine and midwifery, DTU, 2018 (N=42)

S.no	Item	SDA n(%)	DA n(%)	N n(%)	A n(%)	SA n(%)	Mdn (IQR)
**Construction of Professional knowledge**		**1.5(3.5)**	**5(10.7)**	**1(1.7)**	**16(39.3)**	**19(44.6)**	**4.25** **(1.00)**
1	Activate their prior knowledge	0	0	3(7.1)	20(47.6)	19(45.2)	4.00 (1)
2	Interpret, analyze, and apply key concepts precisely	0	3(7.1)	0	21(50)	18(42.9)	4.00 (1)
3	Understand knowledge in-depth	3(7.1)	6(14.3)	0	17(40.5)	16(38.1)	4.00 (1)
4	Gain and retain knowledge for a long period	3(7.1)	9(21.4)	0	8(19)	22(52.4)	5.00 (3)
**Development of Problem-solving skills**		**2 (4.7)**	**2 (4.7)**	**2 (5.5)**	**19 (45.2)**	**17 (39.6)**	**4.00** **(1.00)**
5	Solve real-world problems	0	6(14.3)	2(4.8)	17(40.5)	17.40.5)	4.00 (1)
6	Consider alternatives when solving a problem	3(7.1)	0	5(11.9)	20(47.6)	14(33.3)	4.00 (1)
7	Make a reasonable conclusion	3(7.1)	0	0	20(47.6)	19(45.2)	4.00 (1)
**Development of Self-directed learning**		**0**	**3 (7.1)**	**3 (6.5)**	**20 (47.6)**	**16 (38.7)**	**4.00** **(1.00)**
8	Identify their knowledge gaps	0	3(7.1)	0	23(54.8)	16(38.1)	4.00 (1)
9	Identify and use a variety of resources	0	6(14.3)	3(7.1)	12(28.6)	21(50)	4.50 (1)
10	Think individually or by their own	0	0	5(11.9)	24(57.1)	13(31)	4.00 (1)
11	Plan their work	0	3(7.1)	3(7.1)	21(50)	15(35.7)	4.00 (1)
**Improvement motivation**		**0**	**1.5(3.5)**	**1.5(3.5)**	**22 (51.2)**	**18 (41.6)**	**4.00** **(1.00)**
12	Stimulates students to learn more	0	3(7.1)	0	22(52.4)	17(40.5)	4.00 (1)
13	Stimulates students' interest in learning	0	0	3(7.1)	21(50)	18(42.9)	4.00 (1)
**Promoting group collaboration**		**0**	**0**	**2 (4.7)**	**13 (30.1)**	**27 (65)**	**5.00** **(1.00)**
14	Participate in an open discussion of differing opinions	0	0	3(7.1)	11(26.2)	28(66.7)	5.00 (1)
15	Share what they learned or understood	0	0	3(7.1)	11(26.2)	28(66.7)	5.00 (1)
16	Accept and respect others opinion	0	0	0	16(38.1)	26(61.9)	5.00 (1)
**I prefer PBL to lecture as an effective** **method of learning**		**3(7.1)**	**3(7.1)**	**5(11.9)**	**8(19)**	**23(54.8)**	**5.00(2.00)**

SDA= Strongly Disagree, DA= Disagree, N= Neutral, A= Agree, SA= Strongly Agree, Mdn= Median, IQR= Inter-Quartile Range

Almost all interviewed leaders concluded that PBL is their preferred teaching method. A biomedical unit coordinator said:

“I prefer PBL over other teaching methods in facilitating student learning effectively. Compared to lecture method, I think PBL is the best teaching method.”

**PBL implementation process**: Most students (66.2%) considered the workload imposed by PBL was reasonable, and about 65.4% of the students reported that PBL cases were appropriate and adjusted to students' level of learning. However, only 51.6% of them reported the existence of clear expectations or what to do in PBL sessions.

In DTU, PBL assessment is a continuous assessment of students' performance during tutorials guided by a checklist with regard to content, process and professionalism. The assessment at the end of each session and midway during a module will be used as a formative assessment, while the end-of-module assessment will be used as summative assessment. However, in our study, half of the students did not think the assessment in PBL was appropriate, and about 42.2% of the students reported that it did not consider the improvements made by students with passage of time. Additionally, more than half of students (53.1%) were satisfied with the overall implementation process of PBL (Table 4). Tutors rated the implementation process of PBL more favorably than students. Unlike students, the top two tutors' agreements were on the existence of clear expectations or what to do in PBL sessions (92.8%) and the appropriateness of PBL assessment. Similar to students, most tutors considered the workload imposed by PBL was reasonable (63.6%), and PBL cases were appropriate (64.2%). Additionally, tutors' satisfaction on the overall implementation process of PBL was higher than students' rating (71.4%) (Table 5).

**Table 4:** Students' assessment of PBL implementation process in the department of medicine and midwifery, DTU (N=308)

s.no	Question	SDA n(%)	DA n(%)	N n(%)	A n(%)	SA n(%)	Mdn (IQR)
**Clear objectives/expectations**		**44(14.2)**	**54(17.6)**	**51(16.4)**	**106(34.4)**	**53(17.2)**	**3.50(2.00)**
1	Orientation on the PBL utorial process was provided before the first session	58(18.8)	59(19.1)	52(16.8)	80(25.9)	59(19.1)	3.00(2)
2	The tutor made it clear right from the start what they expected from students in PBL	42(13.6)	63(20.4)	51(16.5)	105(34)	47(15.2)	3.00(2)
3	The work expected or what to do for PBL was known	32(10.4)	41(13.3)	49(15.9)	133(43)	53(17.2)	4.00(1)
**Cases and Composition of** **modules**		**19(6.2)**	**37(12.0)**	**50(16.3)**	**141(45.7)**	**61(19.7)**	**4.00(1.00)**
4	The contents of the PBL cases were adjusted to your level of learning or understanding.	32(10.4)	41(13.3)	27(8.7)	143(46.3)	65(21)	4.00(1)
5	The PBL scenarios were engaging and realistic.	16(5.2)	43(13.9)	56(18.1)	140(45.3)	53(17.2)	4.00(1)
6	The PBL cases integrated knowledge across different disciplines/courses.	16(5.2)	43(13.9)	39(12.6)	151(48.9)	59(19.1)	4.00(1)
7	The PBL cases foster discussion at a higher level of learning	13(4.2)	21(6.8)	79(25.6)	129(41.7)	66(21.4)	4.00(1)
**Workload**		**25(8.1)**	**38(12.2)**	**41(13.4)**	**124(40.1)**	**80(26.1)**	**4.00(2.00)**
8	The schedule of the PBL sessions (2 meetings /week) was adequate to address the objectives.	20(6.5)	32(10.4)	35(11.3)	128(41.4)	93(30.1)	4.00(2)
9	The time given to each session was enough to learn during PBL sessions.	8(2.6)	30(9.7)	24(7.8)	144(46.6)	102(33)	4.00(1)
10	Overall, the workload imposed by PBL was reasonable.	47(15.2)	51(16.6)	65(21)	99(32)	46(14.9)	3.00(2)
**Assessment**		**57(18.4)**	**60(19.4)**	**71(23.2)**	**89(29)**	**31(10)**	**3.00(2.00)**
11	The tutors asked about concepts and mechanisms rather than facts.	17(5.5)	47(15.2)	72(23.3)	135(43.7)	37(12)	4.00(1)
12	The PBL evaluation was both appropriate and fair.	89(28.8)	66(21.4)	68(22)	56(18.1)	29(9.4)	2.00(3)
13	It considers the improvement made by the students with time	64(20.7)	66(21.4)	74(23.9)	77(24.9)	27(8.7)	3.00(2)
**Satisfaction**		**VD** **n(%)**	**D** **n(%)**	**U** **n(%)**	**S** **n(%)**	**VS** **n(%)**	**Mdn** **(IQR)**
14	I am satisfied with the process of PBL implementation.	36(11.7)	54(17.5)	54(17.5)	132(42.7)	32(10.4)	4.00(2.00)

SDA= Strongly Disagree, DA= Disagree, N= Neutral, A= Agree, SA= Strongly Agree, Mdn= Median, IQR= Inter-Quartile Range, VD= Very Dissatisfied, D= Dissatisfied, U= Unsatisfied, S= Satisfied, VS= Very Satisfied

**Table 5:** Tutors' assessment of PBL implementation process in the department of medicine and midwifery, DTU (N=42)

s.no	Question	SDA n(%)	DA n(%)	N n(%)	A n(%)	SA n(%)	Mdn (IQR)
**Clear objectives/expectations**		**0(0)**	**1(3.5)**	**1.5(3.5)**	**15(54.7)**	**11(38.1)**	**4.00(1.00)**
1	I know what to do or what is expected from me for implementing PBL	0	0	3(7.1)	22(52.4)	17(40.5)	4.00(1)
2	I make it clear right from the start what is expected from students in PBL tutorial	0	3(7.1)	0	24(57.1)	15(35.7)	4.00(1)
**PBL Cases and Composition of** **modules**		**4(10.1)**	**5(11.9)**	**6(13.7)**	**19(44)**	**8(20.2)**	**4.00(1.00)**
3	The contents of the PBL cases were adjusted to students' level of learning or understanding.	11(26.2)	7(16.7)	7(16.7)	15(35.7)	2(4.8)	3.00(3)
4	The PBL scenarios were engaging and realistic.	3(7.1)	3(7.1)	9(21.4)	25(59.5)	2(4.8)	4.00(1)
5	The PBL cases integrated knowledge across different disciplines/courses.	3(7.1)	7(16.7)	4(9.5)	17(40.5)	11(26.2)	4.00(2)
6	The PBL cases foster discussion at a higher level of learning	0	3(7.1)	3(7.1)	17(40.5)	19(45.2)	4.00(1)
**Workload**		**1(2.4)**	**9(23)**	**5(11)**	**18(43)**	**9(20.6)**	**4.00(1.25)**
7	The schedule of the PBL sessions (2 meetings /week) was adequate to address the objectives.	3(7.1)	9(21.4)	2(4.8)	18(42.9)	10(23.8)	4.00(2)
8	The time given to each session (2 hours/session) was enough to learn during PBL sessions.	0	11(26.2)	0	21(50.0)	10(23.8)	4.00(2)
9	Overall, the workload imposed by PBL was reasonable.	0	9(21.4)	12(28.6)	15(35.7)	6(14.3)	3.50(1)
**Assessment**		**1(2.4)**	**1(2.4)**	**3(7.1)**	**20(47.6)**	**17(40.5)**	**4.00(1.00)**
10	I provided constructive feedback timely.	3(7.1)	0	0	22(52.4)	17(40.5)	4.00(1)
11	The PBL evaluation was both appropriate and fair.	0	0	6(14.3)	18(42.9)	18(42.9)	4.00(1)
12	The PBL assessment method considers the improvement made by the students with time	0	3(7.1)	3(7.1)	20(47.6)	16(38.1)	4.00(1)
**Satisfaction**		**VD** **n(%)**	**D** **n(%)**	**U** **n(%)**	**S** **n(%)**	**VS** **n(%)**	**Mdn** **(IQR)**
13	I am satisfied with the process of PBL implementation.	3(7.1)	6(14.3)	3(7.1)	15(35.7)	15(35.7)	4.00(2.00)

SDA= Strongly Disagree, DA= Disagree, N= Neutral, A= Agree, SA= Strongly Agree, Mdn= Median, IQR= Inter-Quartile Range, VD= Very Dissatisfied, D= Dissatisfied, U= Unsatisfied, S= Satisfied, VS= Very Satisfied

Academic leaders' response on the appropriateness of PBL implementation and cases was consistent with students' and tutors' ratings. The key informant interviews demonstrated the preparation and hardwork put into the development of quality cases. They said that more than 350 PBL cases had been constructed and revised and the Vice Dean said:

“I believe most of PBL cases were in line with the principles of case development. The HSEDC [Health Science Education Development Centre] office tried to improve cases by forming a multi-disciplinary team even though a few cases need more integration.”

Additionally, interviewed leaders thought the implementation was in line with the principles of PBL, and working on its sustainability was the priority. Midwifery department head mentioned:

“PBL is implemented successfully even if some little gaps happen as we are new for PBL. So I believe these gaps are not major factors that compromise its effective implementation.”

**Students and tutors performance throughout PBL sessions**: Students witnessed the contribution of tutors in enabling them to attain the desired competencies: promoting constructive/active learning skill (71.3%), contextual learning skill (67%) and self-directed learning skill (64.2%) were the top three competencies facilitated by tutors during PBL sessions. However, only 37.6% of students agreed on the intra-personal behaviour of tutors in terms of a motivation to fulfil their role and having a clear picture of his/her strengths and weakness (Table 6). Most tutors for their part agreed that students demonstrated the desired learning outcomes: constructive/active learning skills (87.5%), critical appraisal skills (81%) and collaborative learning skills (78.6%) were the top three learning skills demonstrated by students during PBL sessions.

**Table 6:** Students' evaluation of tutors performance throughout PBL sessions in the department of medicine and midwifery, DTU, 2018 (N=308)

s.no	Items	SDA n(%)	DA n(%)	N n(%)	A n(%)	SA n(%)	Mdn (IQR)
**Constructive/active learning**		**14(4.5)**	**29(9.4)**	**45(14.7)**	**153(49.7)**	**67(21.6)**	**4.00(1.00)**
1	Encourage students to express their own opinions	23(7.4)	30(9.7)	38(12.3)	161(52.1)	56(18.1)	4.00(1)
2	Encourage students to understand underlying mechanisms/theories	11(3.6)	30(9.7)	58(18.8)	144(46.6)	65(21)	4.00(1)
3	Invite students to provide the reasoning behind their opinions	8(2.6)	27(8.7)	40(12.9)	154(49.8)	79(25.6)	4.00(1)
**Self-directed learning**		**16(5.3)**	**34(11.1)**	**60(19.4)**	**142(46.1)**	**56(18.1)**	**4.00(1.00)**
4	Encourage students to generate clear learning issues by themselves	22(7.1)	49(15.9)	54(17.5)	123(39.8)	60(19.4)	4.00(1)
5	Encourage students to search for various resources by themselves	12(3.9)	20(6.5)	57(18.4)	156(50.5)	63(20.4)	4.00(1)
6	Invite students to discuss by guiding from a side rather than giving you the answer	15(4.9)	34(11)	68(22)	147(47.6)	44(14.2)	4.00(1)
**Contextual learning**		**11(3.6)**	**29(9.4)**	**62(20)**	**154(50.1)**	**52(16.9)**	**4.00(1.00)**
7	Support students to apply knowledge on the given problem	14(4.5)	28(9.1)	47(15.2)	165(53.4)	54(17.5)	4.00(1)
8	Support students to apply knowledge to other situations/problems	8(2.6)	30(9.7)	76(24.6)	144(46.6)	50(16.2)	4.00(1)
**Collaborative learning**		**27(8.6)**	**42(13.5)**	**68(22.1)**	**133(43.1)**	**38(12.6)**	**4.00(1.00)**
9	Provide constructive feedback about your group work	30(9.7)	41(13.3)	71(23)	127(41.1)	39(12.6)	4.00(1)
10	Provide constructive feedback about your contribution	36(11.7)	56(18.1)	67(21.7)	122(39.5)	27(8.5)	3.00(2)
11	Invite students to participate in discussion more evenly	14(4.5)	28(9.1)	66(21.4)	149(48.2)	51(16.5)	4.00(1)
**Intra-personal behavior as a** **tutor**		**57(18.5)**	**65(21.1)**	**70(22.7)**	**86(27.9)**	**30(9.7)**	**3.00(2.00)**
12	Has a clear picture of his/her strengths and weaknesses	64(20.7)	72(23.3)	69(22.3)	71(23)	32(10.4)	3.00(2)
13	Motivated to fulfill his/her role as a tutor	50(16.2)	58(18.8)	71(23)	101(32.7)	28(9.1)	3.00(2)

SDA= Strongly Disagree, DA= Disagree, N= Neutral, A= Agree, SA= Strongly Agree, Mdn= Median, IQR= Inter- Quartile Range

**Factors that facilitate PBL implementation**: Key informant interviews with academic leaders identified factors that facilitate PBL implementation related to students, tutors, curriculum, institutional (infrastructure and human resource) and programmatic issues (coordination and monitoring).

**Students and tutors**: Students' and tutors' acceptance of PBL and a strong commitment to their roles, particularly tutors' willingness to be engaged in case preparation and review, were the major opportunities for effective implementation. The Vice Dean of the college reported:

“It is obvious that students were committed to share what they know, discuss together, provide constructive feedback and prepare adequately in a learning issue constructed. This contributed highly for effective implementation of PBL.”

**Curriculum**: Respondents acknowledged the role of the curriculum in providing a contractual agreement between tutors, students and institutions. They emphasized the contribution of the curriculum in integrating different disciplines and providing the type of cases for all weeks. The following extract from social and public health unit coordinator yielded:

“*The curriculum has a great contribution for running PBL as a guiding frame in integrating and providing cases as well as schedules by adjusting the compositions of modules with students level of learning*.”

**Infrastructure**: Respondents described that the college provided adequate resources like small group rooms, charts, markers and whiteboards. A biomedical unit coordinator said:

“*I think the college provides adequate resources for PBL implementation. But still internet service is scarce*.”

**Human resource**: Hiring more tutors has been a priority, with more than 250 instructors employed by the college. Furthermore, PBL implementation started after providing training for the existing instructors and continued for newly employed staff. Since the introduction of PBL, almost all (250 employed academic staff) have been trained. The college Vice Dean reflects the same:

“… almost all newly hired staff have been trained in PBL before involving them in case preparation and tutoring which contributes more for its effective implementation.”

**Coordination**: Smooth coordination starts within the department where department heads played the role of assigning tutors and providing ready-printed cases. PBL coordinators at the department and college level oversee the implementation. That being said, coordinating multidisciplinary teams for case review and development needs to b e strengthened.

**Monitoring**: The college established an office called Health Science Educational Development Centre (HSEDC) mainly responsible for assuring quality education by identifying gaps and intervening in collaboration with departments and the college. The following extract from the college Vice Dean indicates:

“HSEDC is responsible for assuring the quality of cases and implementation process…tutoring. Additionally, there is a PBL committee selected from all departments under HSEDC structure that identified gaps and provided interventions accordingly.”

**Factors that impede PBL implementation**: Even though all interviewed key informants did not mention any barrier, most students listed heavy workload (73.1%) and insufficient time for self-study (67.6%) as major barriers in the self-administered questionnaire. Most tutors for their part described inadequate incentive (81%) and frequent change of leadership (57.1%) in the college as major barriers for PBL implementation.

## Discussion

This study provides the first local evidence on PBL from the perspective of students, tutors and academic leaders. Students, tutors and academic leaders evaluated PBL favorably. It was deemed effective in promoting student learning. Its implementation was considered consistent with the principles of PBL. Students rated tutors' performance positively, and tutors also rated student learning affirmatively. Respondents identified facilitating factors related to students, tutors, curriculum, infrastructure, human resources and program coordination and management. The major barriers were heavy workload and insufficient time for self-study from students' standpoint and lack of incentives and frequent leadership turnover from tutors standpoint.

In our study, students, tutors and academic leaders preferred PBL and perceived it as an effective teaching method in facilitating students' learning in terms of knowledge construction, developing problem-solving and self-directed learning skills, enhancing group collaboration and improving students' motivation to learn. Different studies conducted among students (12,13,16) and tutors (17,18) reported similar results on the perceived effectiveness of PBL.

In this study, the implementation process of PBL was characterized by the existence of clear objectives/expectations, appropriate PBL cases, and reasonable workload. Studies in Korea (19), Saudi (13) and Egypt (20) have reported students' agreement with appropriateness of problem scenarios and the workload. However, students did not think PBL assessment was appropriate which could be related to the absence of formative assessment that consider the improvement of students' performance through time. Another study in Saudi has also reported students' displeasure with the PBL assessment (14). Despite the fact that students should know the work expected or what to do in PBL session, almost half of the students disagreed on the existence of clear objective/expectation. This implied that adequate orientation on PBL tutorial process should be given for students before their exposure to the first session and tutors are expected to make clear what is expected from students in PBL tutorials.

Additionally, in our study, students rated tutors' performance positively and tutors also rated student learning affirmatively. However, only 37.6% of the students agreed on the intrapersonal behavior of tutors characterized by understanding his/her strengths and weakness. This indicated that tutors should develop a habit of providing and receiving feedback at the end of PBL sessions.

In our study, the factors that facilitate PBL implementation were students' and tutors' preferences and commitment to their roles, the contribution of the curriculum in integrating different disciplines and providing the content and schedule of PBL, and institutional leadership ensuring provision of adequate infrastructure, hiring more faculty members, continuous faculty development, strong coordination and the existence of monitoring mechanism. This was consistent with a study done in Malawi (21) and implementation of PBL at a medical school in Ghana faced challenges arising from curriculum design, resource limitations, tutors and case scenarios (22).

From the students' side, heavy workload and insufficient time for self-study and from tutors' side lack of incentives and frequent leadership turnover were the major barriers of PBL implementation. These views are in agreement with results of other studies conducted among students in Egypt (11) and tutors in Nigeria (23).

One important limitation of this study is that we did not evaluate attainment of the desired learning outcomes objectively to speak more confidently about the effectiveness of PBL in improving student learning. The other weakness is that our study is limited to one institution and may not be generalized to other institutions.

In conclusion, our study supports the introduction of problem-based learning in Ethiopia. Students, tutors and academic leaders liked PBL and consider it an effective teaching method. They were satisfied with the overall implementation process and acknowledged the presence of enabling conditions for a successful implementation. However, improving the PBL assessment method and the training or orientation given before starting the PBL sessions have to be strengthened. We recommend that future studies to objectively evaluate attainment of student learning outcomes.
